# Transcriptome Comparison Reveals Key Candidate Genes Responsible for the Unusual Reblooming Trait in Tree Peonies

**DOI:** 10.1371/journal.pone.0079996

**Published:** 2013-11-14

**Authors:** Hua Zhou, Fang-Yun Cheng, Rong Wang, Yuan Zhong, Chaoying He

**Affiliations:** 1 Landscape Architecture College of Beijing Forestry University, National Flower Engineering Research Center, Beijing, China; 2 Institute of Biology and Resources, Jiangxi Academy of Sciences, Nanchang, China; 3 State Key Laboratory of Systematic and Evolutionary Botany, Institute of Botany, Chinese Academy of Sciences, Beijing, China; Institute of Botany, Chinese Academy of Sciences, China

## Abstract

Tree peonies are important ornamental plants worldwide, but growing them can be frustrating due to their short and concentrated flowering period. Certain cultivars exhibit a reblooming trait that provides a valuable alternative for extending the flowering period. However, the genetic control of reblooming in tree peonies is not well understood. In this study, we compared the molecular properties and morphology of reblooming and non-reblooming tree peonies during the floral initiation and developmental processes. Using transcriptome sequencing technology, we generated 59,275 and 63,962 unigenes with a mean size of 698 bp and 699 bp from the two types of tree peonies, respectively, and identified eight differentially expressed genes that are involved in the floral pathways of *Arabidopsis thaliana*. These differentially regulated genes were verified through a detailed analysis of their expression pattern during the floral process by real time RT-PCR. From this combined analysis, we identified four genes, *PsFT*, *PsVIN3*, *PsCO* and *PsGA20OX*, which likely play important roles in the regulation of the reblooming process in tree peonies. These data constitute a valuable resource for the discovery of genes involved in flowering time and insights into the molecular mechanism of flowering to further accelerate the breeding of tree peonies and other perennial woody plants.

## Introduction

The tree peony, which belongs to the genus *Paeonia* L. section *Moutan* DC. (Paeoniaceae), is one of the most important horticultural crops in the world due to its striking ornamental and medicinal values [1], [2]. During its 1600 years of cultivation history, tree peonies were introduced from China to Japan and then to Europe and America; as result, these plants have exerted a tremendous impact and have introduced a flair for the dramatic to gardening [3], [4]. In general, tree peonies flower in early May within a concentrated period of approximately twenty days. Given the commercial value of tree peonies, the achievement of a longer flowering period is the ultimate goal of breeders and growers [5]. Some cultivars have the ability to bloom twice during a given year, which provides a unique opportunity to lengthen the flowering period by exploiting the number rather than the length of the flowering cycles.

Reblooming is an important trait for a variety of horticultural crops that can extend their flowering period and increase their fruit production to thus produce flowers and fruit year-round. In an attempt to force reblooming in tree peonies, various cultivars have been selected, and certain practical techniques, including pruning, gibberellin (GA) treatment, defoliation, and moisture stress, have been considered for a number of years [6]–[8]. To date, tree peonies containing the reblooming trait have become popular in modern gardens and have brought tremendous ornamental and economic successes to China and Japan. A recent study showed that endogenous hormone and carbohydrate levels greatly influence the reblooming process in tree peonies [9]; however, the genetic control of reblooming in this population is not well understood. Molecular biology studies of tree peonies are lacking, although some progresses has been made, including the discovery of some molecular marker [10], [11], the analysis of the effect of chilling on dormancy release [12], and the isolation of the MADS-box genes [13], [14].

Floral transition from a vegetative state to reproductive development in the shoot apical meristem (SAM) marks the beginning of floral initiation and determines the flowering time of a plant [15], [16]. In perennial woody plants, including tree peonies, the juvenile phase of floral transition still poses a challenge for scientists [17]. However, the physiological and molecular genetics of the floral transition in herbaceous species have been extensively studied [18]–[20]. Four pathways controlling the floral transition have been organized in the model plant *Arabidopsis thaliana*. These are the photoperiod and vernalization pathways, which mediate the response to environmental cues, and the autonomous and GA pathways, which are mainly independent from external signals [21]–[23]. The understanding of the floral transition in annual model plants can provide a framework for the exploration this process in perennials.

RNA sequencing (RNA-Seq) is a transcriptome profiling approach that uses deep-sequencing technologies and thus provides a powerful tool for the analysis of species that lack reference genome information [24]. Recent advances in both DNA sequencing and assembly programs have made the low-cost construction of transcriptome datasets for non-model species feasible, which has speeded the process of functional gene discovery [25]–[27]. Many studies have used transcriptome comparisons to identify differentially expressed genes between distinctive plant phenotypes [28]–[31].

In this study, we performed transcriptome sequencing using reblooming and non-reblooming tree peonies to discover potential candidate genes involved in the control reblooming process. To the best of our knowledge, this study provides the first identification of potential flowering genes that are responsible for reblooming in tree peonies and thus serves as a seminal resource for the molecular control of flowering times and for further molecular breeding studies in tree peonies and other perennial plants.

## Materials and Methods

### Plant materials and sample collection

Two cultivars of tree peonies, namelyreblooming *Paeonia lemoinei* ‘High Noon’ (HN) and non-reblooming *Paeonia suffruticosa* ‘Luo Yang Hong’ (LYH), were grown in the Jiu Feng Forestry Experiment Station of the Beijing Forestry University in China. The samples used for sequencing and expression analysis were collected using a binocular microscope to detect the development stage of the flower buds of HN and LYH, which were defined through morphological observation of the SAMs throughout the year using a scanning election microscope (SEM).

Flowers, as well as developing buds, were harvested from HN and LYH for transcriptome sequencing in 2011. The shoot apexes of HN and LYH were collected every three days during floral induction in 2011 and used for quantitative RNA-Seq and real time RT-PCR verification. The following year, shoot apexes, including sprouting reblooming buds (usually big with good nutrition) and non-reblooming buds within scales, were harvested from the same plants from the bud developmental process through the subsequent flowering for gene expression analysis by real time RT-PCR. These samples for real time RT-PCR were collected at approximately 10:00 AM of the sampling day. All of the samples were dissected (i.e., removal of scales and most of the leaves), immediately frozen in liquid nitrogen, and stored at –80°C until further processing.

### Scanning electron microscope (SEM)

The floral induction and differentiation processes were observed through SEM. Prior to analysis, the SAM samples were fixed with 3% glutaraldehyde in 0.025 M sodium phosphate buffer (pH 6.8) at 4°C for 12 hour. The samples were then rinsed in the buffer, further fixed in 1% osmium tetroxide in 0.05 M sodium cacodylate buffer (pH 7.0) for severa1 hours, and then dehydration through a graded ethanol series. The samples were then critical-point dried in liquid carbon dioxide. In many cases, the outer organs were dissected from the SAMs, and the mounted specimens were coated with gold and palladium (4:1) using a Technics Hummer V sputter coater. These studies were performed using an FEI Quanta 200 environmental scanning electron microscope (USA).

### RNA extraction and transcriptome sequencing

The total RNA was isolated using an RN38-EASYspin Plus kit (Aidlab, China).The RNA was then treated with RNase-free I (Promega, USA), and its purity was assessed using a Nanodrop 2000C spectrophotometer (Thermo scientific, USA). After total RNA was collected, the poly (A) mRNA was isolated using Oligo(dT) beads and fragmented into short fragments. The first-strand cDNA was synthesized using random hexamer primers, and the second strand cDNA was synthesized using buffer, dNTPs, RNaseH and DNA polymerase I. These products were purified by agarose gel electrophoresis and enriched by PCR to create the final cDNA library. This library was commercially sequenced by the Beijing Genomics Institute in an Illumina HiSeq™ 2000 platform with paired-end sequencing using 90-bp reads to generate the raw sequences. These data are available at the NCBI Short Read Archive (http://trace.ncbi.nlm.nih.gov/Traces/sra_sub/sub.cgi, accession number: SRP026412 and SRP026299).

### *De novo* assembly and unigene functional annotation

After all of adaptors and low-quality reads were removed from the raw sequences (N > 5%), transcriptome sequences were subjected to *de novo* assembly to form contigs using the short-reads assembling program Trinity [32]. The contigs were further connected to obtain unigenes using the Tgicl clustering tool [33]. The unigenes were divided in to two classes: one was clusters with the prefix CL, and the other consisted of singletons with the prefix unigene. BLASTX alignment and ESTScan was used to predict the coding regions and to determine the sequence direction [34].

For the functional annotation of unigenes, the remaining sequences that could putatively encode proteins were searched against the following protein databases: NCBI non-redundant protein database (NR), Swiss-Prot protein database, Kyoto Encyclopedia of Genes and Genomes (KEGG) database, Gene ontology (GO) database using BLASTX search, and nucleotide database (NT) by BLASTN with e-value less than 0.00001. Based on the NR annotation, the Blast2GO program was used to obtain GO annotation of unigenes [35]. The GO functional classification was analyzed using WEGO software [36].

### RNA quantification and screening of differentially expressed genes

RNA-Seq quantification was performed during floral induction using the same protocol that was used for the transcriptome sequencing except that the RNA-Seq quantification involves single-end sequencing with 50-bp reads. All of the clean reads were mapped to the reference transcriptome sequences (all unigenes) using the SOAPAligner/soap2 program, and the final alignment contained no more than two mismatched base pairs [37].

The gene expression level was calculated using the Reads Per Kilobase per Million mapped reads (RPKM) method [38]. Based on “the significance of digital gene expression profiles”, differentially expressed genes (DEGs) between samples and their corresponding *P*-value were determined using methods described by Audic and Claverie [39]. The False Discover Rate (FDR) was used determine the threshold of the P-value in multiple tests. We used "FDR ≤ 0.001 and the absolute value of log_2_Ratio ≥ 1" as the threshold to judge the significance of the gene expression differences [40].

### Real time RT-PCR verification and expression analysis

The RNA extracted from shoot apex from all of the samples during different floral initiations in HN and LYH in 2011 and 2012 was used for the reverse transcriptase (RT) reaction. The eight specific primer pairs used for real time RT-PCR analyses are shown in Table S1. The PCR was performed on a Mini option Real-Time PCR instrument (Bio-Rad, USA) using 20 µL Taq™ (TaKaRa, Japan). The PCR program was initiated at 95°C for 30 s and was followed by 40 cycles of 95°C for 5 s, 55°C for 30 s, and 72°C for 30 s. A melting curve analysis was then performed for each reaction. The expression levels of the candidate genes were calculated using the 2^−ΔΔCt^ method and normalized to the reference gene *UBIQUITIN* and *GAPDH* [41]. The real time RT-PCR reaction was performed in three biological replicates, and three technical repetitions were performed for each replicate. Expression differences between samples were analyzed by analysis of variance (ANOVA) using SPSS 18.0 software.

## Results

### Morphological description of floral initiation in HN and LYH

**Figure 1:**
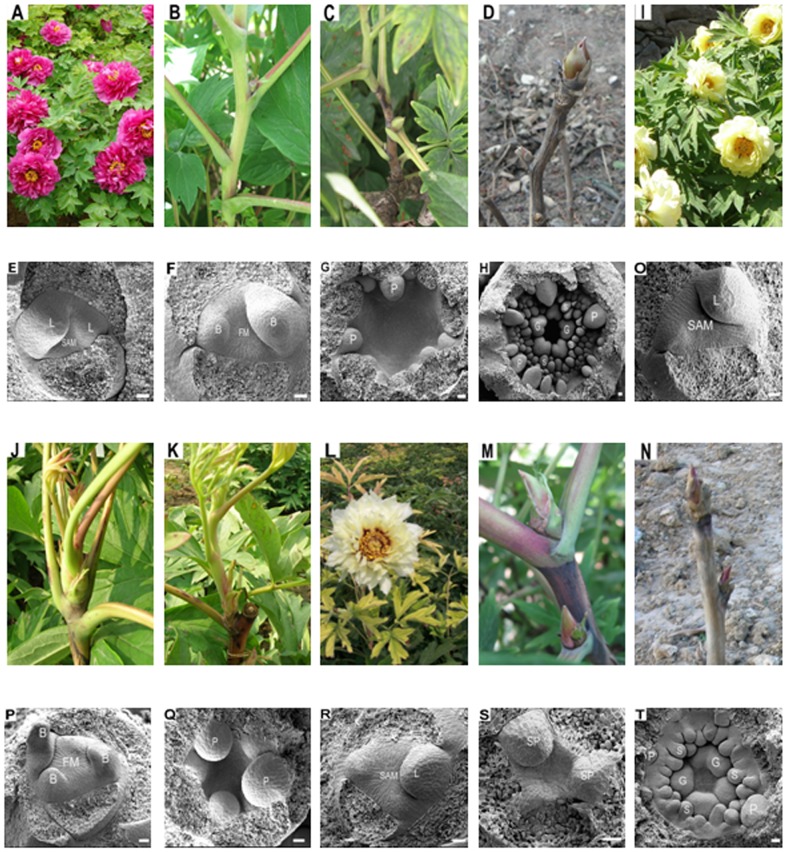
Blooming modes and morphology in LYH and HN throughout the year 2011. These analyses were performed through SEM. LYH: (A) Flowers on 1 May. (B) Lateral buds on 4 July. (C) Lateral buds on 2 September. (D) Dormant apical and lateral buds on 11 November. (E) Leaf primordia were formed on the flanks of the flat and narrow SAM of a bud in May. (F) The meristem enlarged, and bract primordia became visible in July. (G) The SAM became hollow, and pistal primordia arose in September. (H) All of the floral primordia were formed in November. HN: (I) Flowers on 20 May. (J) Outgrowth of annual terminal buds on 9 June. (K) Rapid floral initiation of lateral buds on 2 July. (L) Reblooming on 15 August. (M) Lateral buds without sprouting on 5 September. (N) Dormancy buds on 26 November. (O) Leaf primordia arose in the SAM in May. (P) Bract primordia arose from the floral meristem of outgrowth buds in early July. (Q) Pistal primordia arose from the SAM of the outgrowth buds in late July. (R) Bract primordia were rising from the enlarged SAM in non-sprouting buds in August. (S) Sepal primordia arose in the non-sprouting buds in September. (T) Most floral primordia were formed in November. SAM: shoot apical meristem; L: leaf primordia; B: bract primordia; FM: floral meristem; SP: sepal primordia; P: petal primordia; S: stamen primordia; G: pistil primordia. Bar  =  50 µm.

The ‘High Noon’ (HN) cultivar of tree peonies has been shown to have a stable reblooming trait under cultivation in different countries. As a control, the non-reblooming ‘Luo Yang Hong’ (LYH) cultivar, which is one of the most popular and widely cultivated species in China, was used. Under natural conditions after the winter period, axillary buds give rise to a shoot that is terminated by a flower. Then, in most tree peonies, such as LYH, secondary shoots and annual terminal shoots undergo floral induction in June and then floral differentiation surrounded by bud scales until entering the dormant winter season (Figure 1 A-D). The corresponding histological changes that occur during floral induction and differentiation in LYH were assessed using SEM and are shown in Figure 1 E-H.

The flowering in HN occurs later than in LYH. In this cultivar, floral induction generally takes place in August and followed by floral differentiation within floral buds and then dormancy (Figure 1 M-N). However, some annual terminal shoots and secondary shoots in HN can sprout early in the year; these tend to exhibit rapid floral induction and differentiation and rebloom within a year (Figure 1 J-L). The histological observations during the floral process in HN are shown in Figure 1 O-T. For *Paeonia*, the bracts initiation reveals the end of the vegetative stage and marks the floral transition [42], [43]. The results clearly show one floral transition in LYH (Figure 1 F) and two floral transitions in HN: one transition occurred in the growth buds and resulted in reblooming (Figure 1 P), and the other transition occurred within bud scales to introduce the next spring flowering (Figure 1 R).

The morphological observations ascertained that there is a distinct difference between reblooming and non-reblooming tree peonies and demonstrated that early floral transition and rapid differentiation are critical events in the reblooming cultivar. Therefore, we performed floral transcriptome sequencing between HN and LYH cultivars to further understand these differences at the molecular level.

### Transcriptome sequencing of HN and LYH and *de novo* assembly

We constructed cDNA libraries for both HN and LYH. Total of 55,245,430 and 57,483,678 raw 90-bp reads were separately generated on an Illumina HiSeq™ 2000 instrument (Table 1). The primer and adaptor sequences were removed to generate clean reads, which were then subjected to cluster and assembly analysis. The software assembled 150,407 and 132,499 contigs with a mean length of 320 bp and 334 bp for HN and LYH, respectively, and obtained 63,962 and 59,275 unigenes with a mean size of 699 bp and 698bp, respectively (Table 2). Altogether, 84,919 unigenes were clustered from the two cultivars, and 33,500 and 51,419 of these clusters and singletons, respectively (Table 2). The size distribution of the assembled contigs and unigenes is presented in Table S2.

**Table 1:** Summary of HN and LYH transcriptome sequencing

Sample	Total raw reads	Total clean reads	Total clean nucleotides (nt)	Q20 percentage	N percentage	GC percentage
HN	55,245,430	51,411,776	4,627,059,840	97.23%	0.00%	45.87%
LYH	57,483,678	54,163,770	4,874,739,300	97.99%	0.00%	46.13%

**Table 2:** Summary of HN and LYH transcriptome assembly

	Sample	Total number	Total length (nt)	Mean length (bp)	N50	Clusters	Singletons
Contig	HN	150,407	48,120,963	320	590		
Contig	LYH	132,499	44,276,926	334	624		
Unigene	HN	63,962	44,707,077	699	1077	27,452	36,510
Unigene	LYH	59,275	41,380,872	698	1075	19,288	39,987
All Unigene		84,919	58,746,795	692	1093	33,500	51,419

### Transcriptome functional annotation and comparison

To identify the putative functions of the assembled unigenes in HN and LYH, we searched for sequence similarities in five public databases: NR, Swiss-Prot, KEGG, GO database using the BLASTX search and NT by BLASTN. In total, 36,821 of the 63,962 (57.5%) in HN and 36,475 of the 59,275 (61.5%) unigenes in LYH have been annotated in the public databases (Table 3). Of the BLAST hits, most of the unigenes (96.8% in HN and 96.7% in LYH) were annotated by the NR database; the annotated unigenes are listed in Table S3.

**Table 3:** Statistics on the number of unigenes in HN and LYH annotated with the NR, NT, Swiss-Prot, KEGG, and GO databases

Sequences	NR	NT	Swiss-Prot	KEGG	GO	All databases
Unigene in HN	35,657	29,742	21,526	19,135	15,896	36,821
Unigene in LYH	35,283	29,980	21,345	19,257	16,305	36,475
All unigene	41,493	34,293	25,347	22,542	19,338	43,235

**Figure 2:**
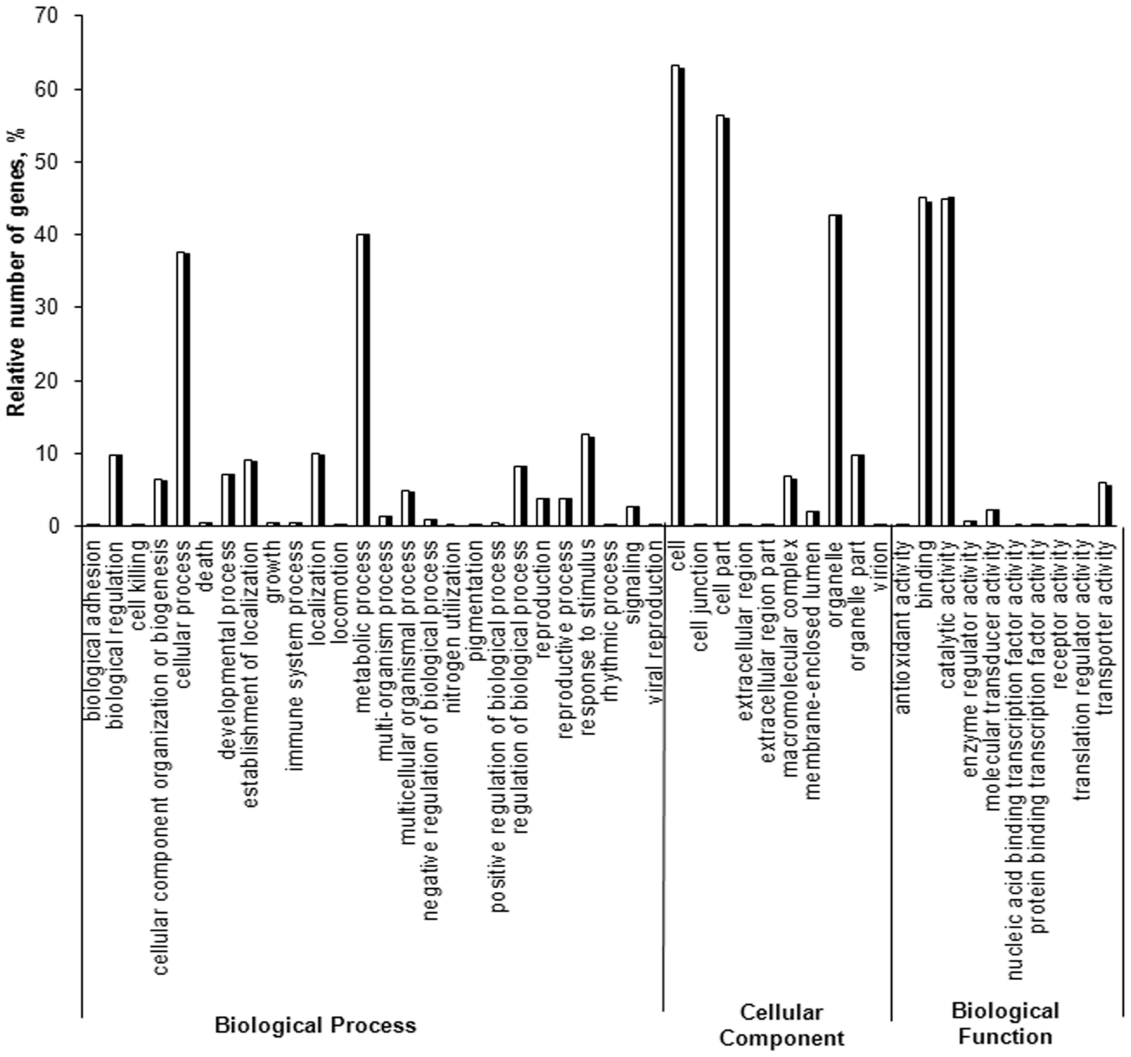
Histogram representation of GO classification for HN and LYH. The results are summarized in three main categories: biological process, cellular component, and biological function.

To understand the differences in the floral initiation process between HN and LYH, their transcriptomes were compared. The GO analysis showed that the distribution of gene functions for the cDNA sequences from HN and LYH were similar in three main categories: biological processes, cellular components, and molecular functions (Figure 2). Among these categories, the cell, cell junction, binding, catalytic activity, organelle, metabolic process and cell process were the groups with the highest representation of genes from both cultivars.

Functional classification and pathway assignments for HN and LYH were performed through KEGG analyses. A total of 19,135 and 19,257 matched sequences of HN and LYH, respectively, were assigned to 127 KEGG pathways. The most represented pathways in HN and LYH are the metabolic pathways (20.56% and 21.13%, respectively), the biosynthesis of secondary metabolites (10.1% and 9.64%, respectively), plant-pathogen interaction (5.99% and 5.55%, respectively), and plant hormone signal transduction pathways (5.38% and 5.16%, respectively; Table S4).

This transcriptome annotation between HN and LYH provides a valuable resource for gene discovery and functional analysis in tree peonies.

### Screening and analysis of differentially expressed genes (DEGs) through quantitative RNA-Seq

**Figure 3:**
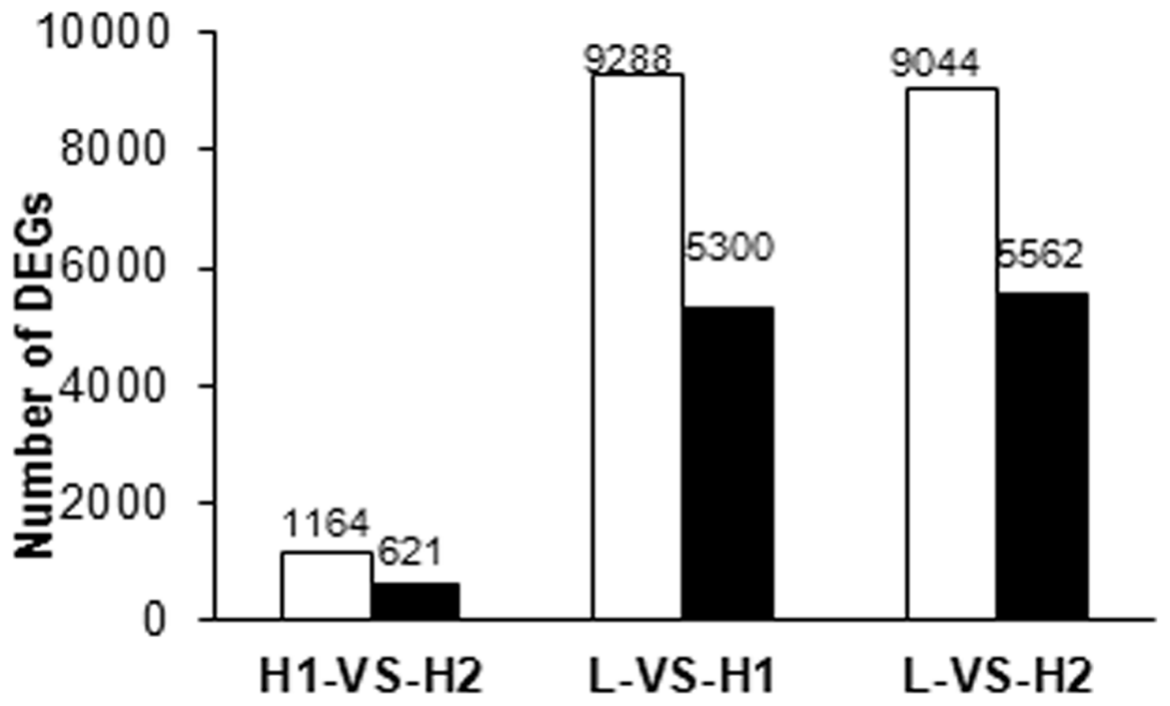
Changes in the gene expression profile among the different floral inductions. The gene expression profiles were determined by RNA-Seq. The changes in the number of up-regulated and down-regulated genes between H1 and H2; L and H1; and L and H2 are summarized. The white box represents the up-regulated genes and the black box represents the down-regulated genes.

The morphological analysis demonstrated that the early floral transition in HN is the primary event that characterizes reblooming. To determine potential genes involved in reblooming induction, we compared the gene expression profiles of HN and LYH during floral transition. Floral induction in tree peonies is defined as the stage when leaf primordial transitions to bract primordial [44]. The exact sampling period in the present work was from 5 June to 25 June 2011 for LYH and for reblooming induction in HN and from 1 August to 25 August 2011 for normal floral induction in HN. Subsequently, the samples collected during floral induction were used to construct three cDNA libraries, namely floral induction in LYH (L), normal floral induction in HN (H1), and reblooming induction in HN (H2), for quantitative RNA-Seq analysis. We generated 12,488,008, 12,380315 and 12,041,487 50-bp reads through Illumina sequencing from the L, H1 and H2 libraries, respectively (Table S5). After removing the adaptor and low-quantity raw reads, we mapped the clean reads of these three libraries to the transcriptome database, which contains 84,919 unigenes. More than 80% of the clean reads in the induction stages were mapped to reference transcriptome sequences, and 58.43%, 58.29%, and 58.37% unique matches were found for these three stages (Table S5). The differentially expressed genes (DEGs) were then identified from the three floral induction cDNA libraries. As expected, the majority of gene expression changes occurred between L and H1/H2, and slightly more up-regulated genes were observed compared with down-regulated genes among the groups (Figure 3 and Table S6, S7, S8).

**Figure 4:**
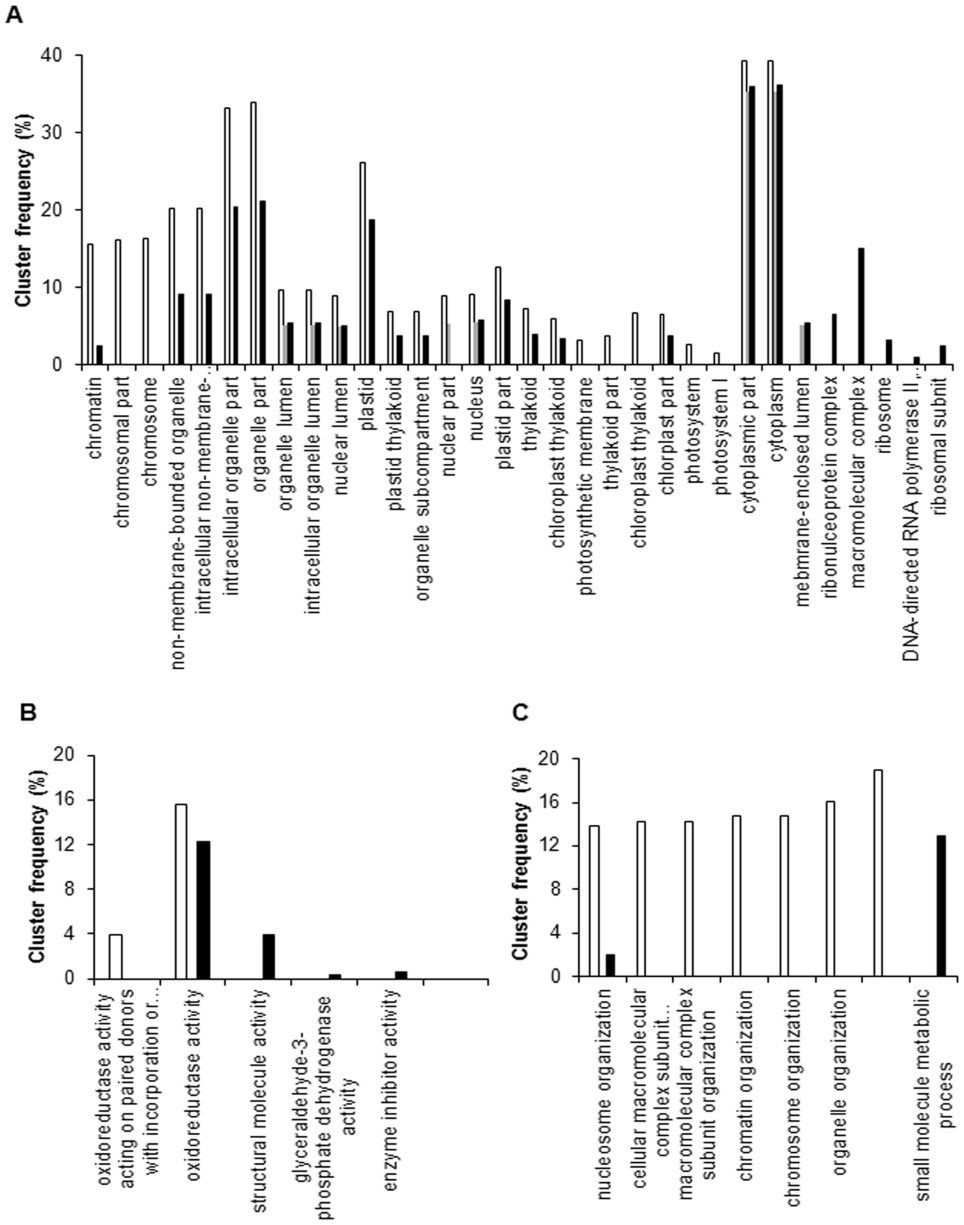
Functional enrichment analyses of DEGs in the GO annotation. The cluster frequency of the GO terms that were significantly enriched (*P*-value < 0.05) were analyzed in the three comparisons (H1-VS-H2, L-VS-H1, and L-VS-H2). The white box represents H1-VS-H2, the gray box represents L-VS-H1, and the black box represents L-VS-H2. A: cellular component; B: molecular function; C: biological process.

GO and KEGG assignments were performed to clarify the functions of the DEGs in the three cDNA libraries during floral induction. Fewer DEGs were classified into the three major GO categories (cellular component, molecular function, and biological process) in L-VS-H1 compared with H1-VS-H2 and L-VS-H2 (Figure 4). In the cellular component category, the ‘intracellular organelle part’, ‘organelle part’, ‘plastid’, ‘cytoplasm’, and ‘cytoplasmic part’ were significantly enriched (*P* < 0.05) in all three induction comparisons (Figure 4 A). In the molecular function category, only one term, namely ‘oxidoreductase activity acting on paired donors’, was significantly enriched in H1-VS-H2 and L-VS-H2, and no term was significantly enriched in L-VS-H1 (Figure 4 B). In the biological process category, most DEGs were significantly enriched in H1-VS-H2, and no term was significantly enriched in L-VS-H1 (Figure 4 C).

In the H1, H2, and L comparisons, 936, 4114, and 4710 DEGs were mapped to 107, 123, and 124 KEGG pathways, respectively. ‘Metabolic pathway’ and ‘biosynthesis of secondary metabolites pathway’ were significantly enriched in the three comparisons (Table S9). In addition to the metabolic pathways, photosynthesis, spliceosome, circadian rhythm in plant, and ribosome pathways were significantly enriched in the different comparisons (Table S9).

### Candidate genes in the floral pathway from DEGs

Based on the functional annotation, all of the unigenes were analyzed through BLASTX searches, and 21 known *Arabidopsis* homologous genes involved in four floral pathways were identified (Table S10). Of these 21 genes, eight genes were differentially expressed between the three floral induction patterns and may thus be associated with reblooming induction (Table 4).

**Table 4:** Putative candidate genes of the DEGs associated with floral induction in tree peonies

Tree peony gene	*Arabidopsis* ID	Unigene ID	% identity with homologs	RPMK of H1H1-VS-H2FDR	RPMK of H2L-VS-H1FDR	RPMK of LL-VS-H2FDR
Photoperiod pathway						
PsCO	NP_568863.1	CL8074.Contig2_All	47	17.3−0.470.027	12.57.558.65E-54	0.097.082.86E-38
*PsGI*	NP_564180.1	CL9113.Contig1_All	75	2.24−0.030.98	2.194.041.32E-14	0.134.03.14E-14
Vernalization pathway						
*PsFRI*	NP_850923.1	CL10369.Contig1_All	63	48.3−1.029.45E-29	23.8−0.836.94E-36	85.7−1.851.1E-124
*PsVIN3*	NP_200548.2	Unigene3778_All	41	12.760.756.16E-07	21.50.730.00017	7.681.481.97E-20
GA pathway						
*PsGA20OX*	NP_194272.1	Unigene3745_All	52	7.81−1.170.00067	3.47−0.350.21	9.97−1.521.71E-07
*PsGID 1*	NP_187163.1	Unigene2987_All	81	243.4−1.840	67.80.252.23E-10	204.7−1.65.26E-211
Floral integrators						
*PsSOC1*	NP_182090.1	Unigene15356_All	61	19.11.42.64E-22	50.6−1.335.68E-20	48.20.0670.63
*PsFT*	NP_176726.1	Unigene5093_All	78	1.212.474.51E-06	6.731.950.15	0.314.423.14E-10

H1-VS-H2:log_2_ gene expression level in H2 compared to H1.

A FDR (false discovery rate) < 0.001 indicates a significant difference.

Note: percent identity was calculated by comparing the *Arabidopsis* and tree peony sequences at the amino acid level.

The analysis of the autonomous pathway showed that most of the homologous gene*s* of *Arabidopsis* were present in tree peonies [45]. The expression of these genes, although slightly variable, exhibited no significant difference among the three floral inductions (Table S10).

An analysis of the photoperiod pathway using the homologous genes in *Arabidopsis* [46] demonstrated that certain photoperiod response genes exhibited a higher expression level in HN compared with LYH, although not all of the genes were significantly differentially expressed (Table S10). *CO* (*CONSTANS*) [47] and *GI* (*GIGANTEA*) [48], which are two important photoperiod genes, showed approximately seven- and four-fold higher expression levels in H1 and H2 than in L (Table 4). In the vernalization pathway [49], only two homologous genes, namely *FRI* (*FRIGIDA*) [50] and *VIN3* (*VERNALIZATION INSENSITIVE 3*) [51], were found. *PsFRI*, which is a floral repressor, and *PsVIN3*, which is a flowering enhancer, presented converse expression in H2 compared to H1 and L (Table 4). Of the four homologous genes found in the GA-signaling pathway [52], *GA20OX* (*GIBBERELLIN 20OXIDASE)*, which is a key gene responsible for active GA synthesis [53], and *GID1* (*GA INSENSITIVE DWARF 1*), which is an active GA receptor [54], exhibited similar down-regulation in H2 compared to H1 and L (Table 4).

In addition to the four floral pathways, we searched for homologs for the floral pathway integrator *SOC1* (*SUPPRESSOR OF CONSTANS OVEREXPRESSION 1*), *FT* (*FLOWERING LOCUS T*), *LFY* (*LEAFY*), and the meristem identity gene *AP1* (*APETALA 1*) in *Arabidopsis* (Table S10) [55], [56]. Through this analysis, the *SOC1* [57] and *FT* [58] homologous genes were shown to be differentially expressed between the different floral inductions (Table 4).

In general, eight DEGs, namely *PsCO*, *PsGI*, *PsFRI*, *PsVIN3*, *PsGA20OX*, *PsGID1*, *PsSOC1* and *PsFT*, were found to be candidate genes associated with reblooming induction. To date, no function has been reported for any of these genes in tree peonies, although two of these genes, *PsGA20OX* (GenBank ID: ABQ52488.1) and *PsAP1* (GenBank ID: ADM21461.1), are registered in the NCBI with unknown gene function.

### Expression analysis of candidate genes during the flowering process in HN and LYH

**Figure 5:**
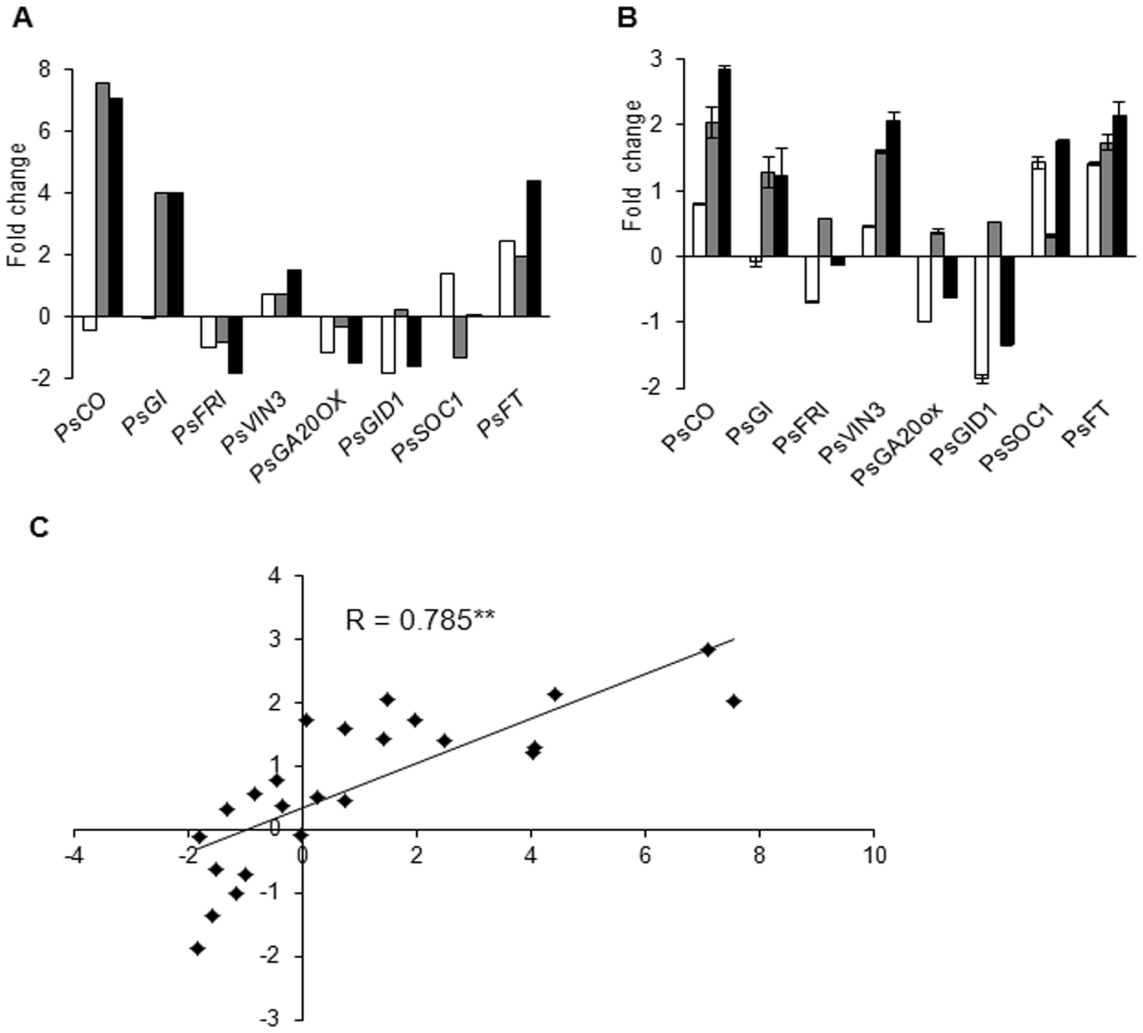
Gene expression changes during floral induction. (A) Gene expression data obtained through RNA-Seq analysis. (B) Real time RT-PCR analyses of gene expression ratios. The white box represents gene expression changes between H1 and H2. The grey box represents gene expression changes between L and H1. The black box represents gene expression changes between L and H2. The y-axis represents the fold change of the gene, which was calculated using the log_2_ value of each induction stage. The bars represent the standard error (n  =  3). (C) Pearson correlation analysis of the gene expression ratios obtained from the RNA-Seq and the real time RT-PCR data. The real time RT-PCR log_2_ values (expression ratios; y-axis) were plotted against the RNA-Seq log_2_ values (x-axis). The Pearson correlation coefficient (R) is given in the plot, and the asterisks indicate the extreme significant difference at *P* < 0.01.

To verify the accurateness of the RNA-Seq data (Figure 5 A), we validated the candidate DEGs through real time RT-PCR. As shown in Figure 5 B, the expression levels of these genes are varied among the three floral induction stages, and the expression pattern of these genes was similar to those obtained through RNA-Seq, which confirms the validity of the RNA-Seq data. A Pearson correlation analysis between the gene expression levels measured by real time RT- PCR and RNA-Seq showed a highly significant correlation (correlation coefficient R  =  0.785, *P* < 0.01), which indicates good reproducibility between the transcript abundance assayed by RNA-Seq and the expression profile revealed by real time RT-PCR (Figure 5 C).

**Figure 6:**
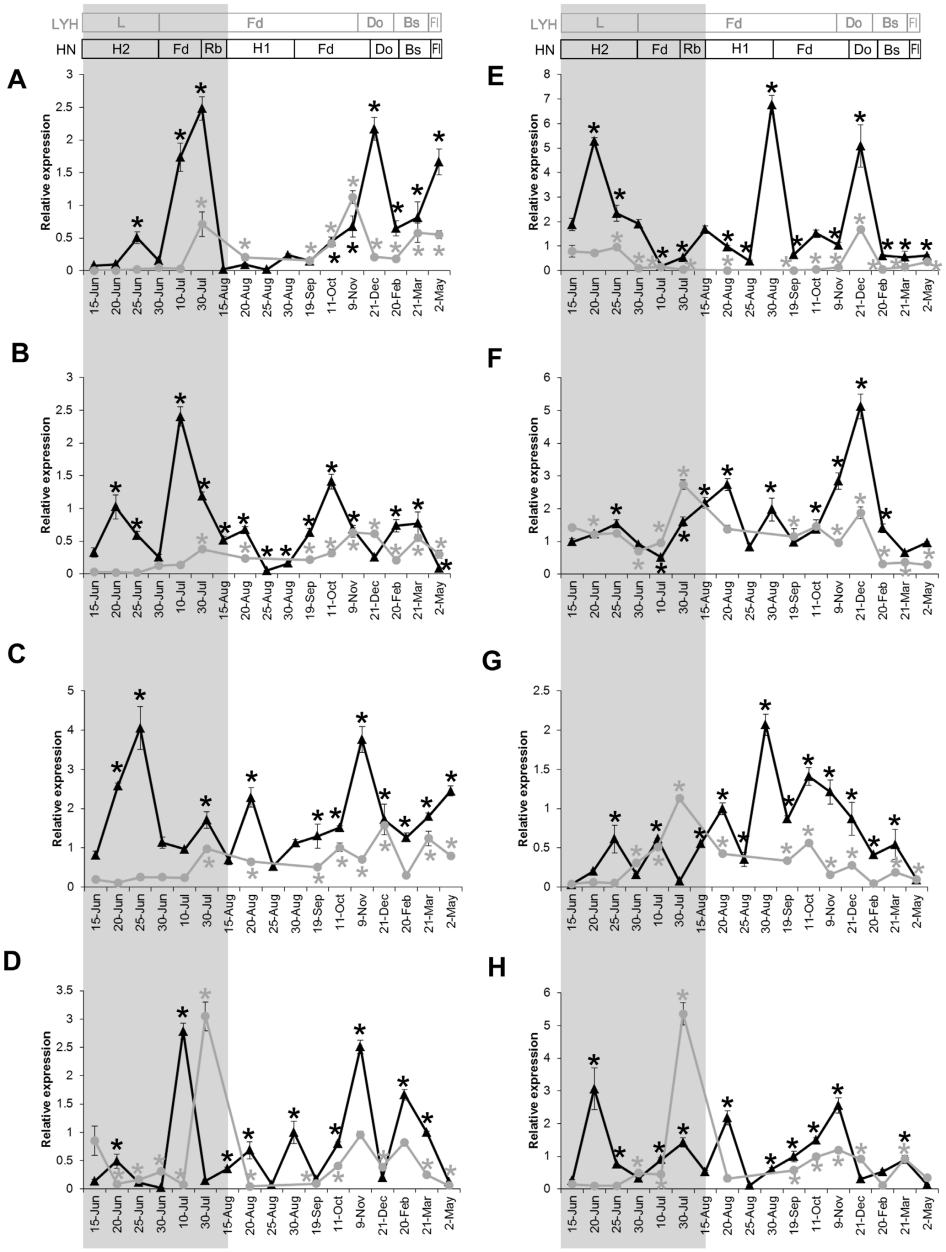
Expression patterns of candidate genes in HN and LYH over the period 2012–2013. The expression changes of candidate genes: *PsFT* (A), *PsVIN3* (B), *PsCO* (C), *PsGA20OX* (D), *PsSOC1* (E), *PsFRI* (F), *PsGID1* (G), and *PsGI* (H). The x-axis indicates the dates at which the shoot apexes were harvested from HN (triangles in black line) and LYH (circles in gray line) in 2012–2013. The gene expression levels are expressed relative to the first sample for each gene. The bars represent the standard error (n  =  3). After the spring flowering in 2012, the shoots of LYH underwent floral induction from 15 June to 30 June (L) and then entered floral differentiation and dormancy. In 2013, the bud sprouted in late February and flowered in early May in LYH. In HN, the buds underwent reblooming induction from 15 June to 30 June (H2), followed by rapid floral differentiation, and reblooming. The non-sprouting buds in HN underwent floral induction from 15 August to 30 August (H1) and then underwent differentiation and dormancy. The buds sprouted in early March and flowered in early May in 2013. The floral induction and differentiation stages throughout the year were defined through a histological analysis, as described in Figure 1. Only one sample was detected in August in LYH. For each gene, gene expression level in HN and LYH was present as mean of three biological replicates and the statistical analysis was realized through one-way analysis of variance, and the asterisk represents a significant difference at *P* < 0.05. The significance was compared relative to the first sample from HN and LYH, respectively. Fd: floral differentiation; Do: dormancy; Bs: bud sprouting; Fl: flowering; Rb: reblooming. L: floral induction in LYH; H1: normal floral induction in HN; H2: reblooming induction in HN.

Through our study of floral induction, we discovered eight differentially expressed genes that are associated with reblooming. To further confirm the role of these genes in reblooming, we analyzed their change in gene expression during floral initiation in HN and LYH by real time RT-PCR in 2012–2013 (Figure 6).

The analysis of the floral development process showed that the *PsFT* expression was low during induction and then increased significantly during differentiation in both HN and LYH (Figure 6 A). The statistical analysis showed that *PsFT* decreased significantly during the two floral inductions (H2 and H1) in HN and only once (L) in LYH (Figure 6 A). The *PsVIN3* gene presented a high expression level during reblooming differentiation in July and decreased during normal differentiation in HN (Figure 6 B). The *PsCO* gene exhibited a high expression level during reblooming induction and normal differentiation in HN (Figure 6 C). Similarly, the *PsVIN3* and *PsCO* genes presented the same expression pattern as *PsFT* in LYH, which was significantly accumulated during differentiation (Figure 6 A, B, C). The *PsGA20OX* gene showed fluctuation during the floral process in HN and LYH and accumulated during differentiation (Figure 6 D).

Certain genes, including *PsSOC1*, *PsFRI* and *PsGID1,* exhibited a different expression pattern with high expression during the normal floral transition in HN (Figure 6, E, F, G). In contrast to *PsFT*, the expression of *PsSOC1* gene was high during induction, decreased during differentiation, and increased again during bud dormancy; this pattern was consistent in the three floral initiation periods (Figure 6 E). The expression of the *PsFRI* gene, which is the only floral repressor in our list of candidate DEGs, was high during floral induction and differentiation and decreased significantly after bud burst in LYH; in contrast, this gene exhibited a significant low expression during reblooming differentiation, and its level was accumulated during bud dormancy in HN (Figure 6 F). The *PsGID1* gene was accumulated early during the differentiation processes and decreased from October to February in both HN and LYH (Figure 6 G).

The *PsGI* gene demonstrated a similar expression pattern as *PsCO* throughout the floral developmental process in HN but exhibited higher accumulation during floral differentiation in 30 August in LYH compared with HN (Figure 6 C, H).

## Discussion

### Reblooming in tree peonies: a distinctive floral initiation

Reblooming is a common phenomenon in horticultural crops and is one way to efficiently extend the flowering period. There are two conditions for reblooming during the same year for perennial plants: one can flower continuously during the favorable season, and the other may only have a second bloom later in the season [59]. Most horticultural crops, such as some fruit trees, belong to the latter due to the break out of resting buds. In this study, we analyzed the morphological modifications that occur during the reblooming process in HN. The results showed that the reblooming in HN may be distinct from other reblooming processes. First, two floral transitions during two concentrated periods take place in HN: the first transition in June results in reblooming, and the second transition in August introduces the flowering for the following spring. However, in some fruit trees, reblooming has only one floral transition, and some environment conditions induce buds to break and to thus flower earlier than dormancy buds [60]. Second, floral induction and differentiation during reblooming in HN are accompanied by bud sprouting and growth (Fig. 1 J, K, P, Q). In contrast, bud breakage usually occurs in non-sprouting dormancy buds that have partly or completely differentiated their floral primordial [61].

The timing of plant flowering varies greatly by genotype and is highly dependent on genetic and environmental interactions. In roses, continuous flowering is self-inductive and is not dependent on environmental control, whereas the floral induction of single flowering plants is dependent on environmental control [62]. In fact, conflicting reports state that woodland strawberries have either autonomous control of flowering [63] or long-day rather than autonomous control of continuous flowering [64]. The following genes play key roles in the control of continuous flowering in roses and strawberries: the *TFL1* homologue *KSN* [59], *RECURRENT BLOOMING (RB)* and *SEASONAL FLOWERING LOCUS* (*SFL*) in roses and strawberries [65], [66]. However, the genes identified in rose and strawberry plants do not appear to be the candidate genes for tree peonies, which indicate that separate mechanisms control reblooming in these different plant species.

### A comprehensive floral transcriptome of tree peonies

To identify genes that are involved in the reblooming process in tree peonies, we sequenced the floral transcriptomes from reblooming HN and non-reblooming LYH. We generated a total of 84,919 unigenes with mean reads of 692 bp, which corresponds to a 3.5-fold higher number of unigenes compared with the transcriptome of *Paeonia ostii* ‘Feng Dan’ for which 23,652 contigs/singletons were generated from dormancy buds, which accounts for 15,284 contigs/singletons with an average mean read of 758.9 bp using the Roche 454 GS FLX platform [67]. More than 50% of all of the unigenes were annotated using public databases with key homologous genes involved in the four floral pathways of *Arabidopsis*. These floral transcriptomes of both cultivars provide more comprehensive information and gene resources for the study of the floral transition and development in tree peonies and might facilitate further investigations into the molecular mechanism of flowering in this important crop.

### Candidate genes for the control of reblooming in tree peonies

Based on transcriptome and gene expression analyses of the floral process in HN and LYH, we identified genes that are differentially expressed in these floral pathways and demonstrated that these may play a role in reblooming.

Flowering is controlled by a complex network of signaling pathways. One environmental cue is light. In *Arabidopsis*, flowering is induced in long-day conditions by the *CO* gene, which up-regulates the expression of the *FT* gene [68]. However, no previous reports have described the influence of the photoperiod on flowering in tree peonies. In this study, the *PsCO* gene was accumulated during reblooming induction and exhibited a higher expression level in HN compared with LYH (Figure 6 C, H). The up-regulation of the expression of this gene during reblooming induction coincided with the bud sprouting and outgrowth that occurred in HN in June. The KEGG pathway analysis of the DEGs during floral induction showed that photosynthesis was significant enriched in H1-VS-H2 (3.1% of DEGs) and L-VS-H2 (0.87% DEGs) (Table S9). Therefore, compared to normal flowering, we conclude that light may have a stronger effect on induction of reblooming and bud growth. Given that the constitutive overexpression of *CO* causes rapid flowering even in non-inductive conditions [69], the fact that the *PsCO* gene is associated with reblooming in our study is very interesting and warrants further investigation.

In perennial woody plants, such as tree peonies, chilling is normally required for dormancy release prior to flowering, and this process is similar to vernalization in *Arabidopsis*, where flowering is promoted through exposure to cold temperatures [70]. However, HN blossoms directly without cold vernalization (Figure 1 I-L). In this study, the high expression of the vernalization gene *PsVIN3* and the low expression of *PsFRI* during floral differentiation in 10 July is likely responsible for the direct reblooming in HN. *VIN3*, which is a key gene that responds to vernalization conditions in *Arabidopsis*, is induced by low temperatures and repressed upon a return to warmer temperature [71], [72]. In HN, *PsVIN3* was up-regulated during reblooming induction on 20 June and was abundantly accumulated during reblooming differentiation on 10 July (Figure 6 B). Remarkably, the expression pattern of *PsVIN3* was similar to *PsFT* in both cultivars during floral development (Figure 6 A, B). These results suggest that both *PsVIN3* and *PsFT* most likely play important roles in the promotion of reblooming.

Considerable physiological and molecular analyses have shown that GA plays an important role in the control of floral transition, and its role may be species-specific [73]. In *Arabidopsis,* GA contributes to the floral pathway through the promotion of floral induction; in contrast, GA generally inhibits flowering in woody angiosperms [17], [74], [75]. In this study, a low expression of *PsGA20OX* and *PsGID 1* was detected in all three floral inductions (Figure 6 D, G), which suggests that GA might play a role in tree peonies that is similar to its role in the inhibition floral induction in other woody plants. *PsGA20OX* exhibited very low expression during reblooming induction but was rapidly increased afterward to reach peak abundance (Figure 6 D), which indicates that a low expression of GA may induce reblooming and that a high expression of GA may promote reblooming differentiation. This finding is consistent with the results from other studies, which have demonstrated that exogenous application of GA_3_ to flower buds can help promote flowering in tree peonies. In contrast to *PsGA20OX, PsGID1* was accumulated during normal floral transition in HN (Figure 6 D, G), which is similar to the feedback regulation of *GID1* through the GA signaling pathway in rice and *Arabidopsis* [76], [77]. The expression of the *PsGA20OX* gene fluctuated similarly to the change in the GA_3_ pattern during floral initiation, as was previously reported in tree peonies [9], [78]. This finding leads us to hypothesize that *PsGA20OX* plays an important role in reblooming in tree peonies that is mediated through the GA pathway.

Floral pathway integrators incorporate multiple floral pathways to determine the exact flowering time [79]. In this study, the expression level of the *PsSOC1* gene was initially high and then decreased during floral initiation (Figure 6 E), which indicates that *PsSOC1* might play a role in the promotion of floral transition but has no effect on floral organogenesis in tree peonies. In additional, the level of *PsSOC1* increased significantly again in 21 December, and this change corresponded to a change in the expression of *PsFRI*, which suggests that *PsSOC1* might be associated with chilling requirement and bud dormancy break [80]. *FT,* which is as a major component of florigen, is involved in the regulation of the floral transition in all angiosperms examined to date [81]. In trees, *FT* also controls seasonal growth cessation and dormancy [82], [83]. *PsFT*, which was found to be accumulated through the floral process, demonstrates a conserved role in the promotion of flowering in tree peonies. Notably, the expression of *PsFT* was up-regulated before reblooming, and this increase coincided with the accumulation of *PsVIN3*, *PsCO* and *PsGA20OX* in the sprouting buds (Figure 6 A, B, C, D), which suggests that *PsFT* might be associated with bud growth and that *PsFT* promotes reblooming by integrating different endogenous and environmental signals from the vernalization, photoperiod and GA pathways.

## Conclusions

In conclusion, this study provides the first set of comprehensive floral transcriptome data in tree peonies. The transcriptome comparison between reblooming and non-reblooming tree peonies revealed eight DEGs as potential candidates responsible for reblooming. Detailed expression analyses of these genes during floral transition demonstrated that four DEGs, namely *PsFT*, *PsVIN3*, *PsCO*, and *PsGA20OX* might be responsible for reblooming in tree peonies. These findings offer the first insights into the potential mechanisms underlying flowering and reblooming in tree peonies.

## Supporting Information

Table S1 Primers for real time RT-PCR analysis.
(DOC)

Table S2 Overview of HN and LYH transcriptome sequencing and assembly. (A) Size distribution of Illumina sequencing contigs. (B) Size distribution of Illumina sequencing unigenes after paired-end gap filling.
(XLS)

Table S3 Top BLAST hits from the NCBI NR database. BLAST results against the NCBI NR database for all distinct sequences with e-value < 0.00001.
(XLSX)

Table S4 Pathways represented by the unigenes from HN and LYH.
(XLS)

Table S5 Statistics of the mapping to all of the unigenes in the three floral inductions.
(DOC)

Table S6 Differentially expressed genes between H1 and H2.
(XLS)

Table S7 Differentially expressed genes between L and H1.
(XLS)

Table S8 Differentially expressed genes between L and H2.
(XLS)

Table S9 KEGG pathways that were significantly enriched during the three floral inductions. The KEGG pathways significantly enriched were those pathways with *P* < 0.01.
(XLS)

Table S10 The 21 genes putatively associated with floral induction in HN and LYH.
(DOC)
