# Supplementary material for: Transcriptome Comparison Reveals Key Candidate Genes Responsible for the Unusual Reblooming Trait in Tree Peonies
Source: PLoS One. 2013 Nov 14;8(11):e79996. doi: 10.1371/journal.pone.0079996 (PMC3828231; doi:10.1371/journal.pone.0079996)
Supplement: Table S1 — Primers for real time RT-PCR analysis. (DOC) [file pone.0079996.s001.doc]

**Table S1 Primers for r**eal time RT-PCR analysis

| **Gene** | **Forward prime (5’-3’)** | **Reverse prime (5’-3’)** | **Size of product (bp)** | **Primer efficiency**  **(%)** |
| --- | --- | --- | --- | --- |
| *PsCO* | AGATGCCGAAGAAGAAGCC | CCAGTAAAATCAACCTCAAAGC | 300 | 98.3 |
| *PsGI* | TCCACCGCAAGATACGCA | GGGCAGACTAAGGAAATGAAAG | 288 | 94.6 |
| *PsFRI* | CCCATAAACGCAAGAGGACC | AGAAAGCCAAGGCAGACAAG | 199 | 96.1 |
| *PsVIN3* | AACCCCGAGTATCCCTGAA | CAAGCCTTTGGCTGACATG | 182 | 98.0 |
| *PsGA20OX* | GGCACATGACGAAATGAATG | TGAGTGGGCACCCGAATA | 103 | 103.1 |
| *PsGID1* | AAGCCTTTGAACGCTGACA | TTAAGAGCTGCCCAACCAT | 206 | 95.9 |
| *PsSOC1* | TGCCGAGGTTGCCCTTATC | CCGCTCCTGGTTTCCACATT | 123 | 100．1 |
| *PsFT* | GTGTAGTTGGGGATGTCTTGGA | TTAGGTTTGGGTCGCTTGG | 210 | 102.9 |
| *PsUBIQUITIN* | GACCTATACCAAGCCGAAG | CGTTCCAGCACCACAATC | 142 | 98.6 |
| *PsGAPDH* | GGTTGATCTCACTGTTAGGC | TCAGACTCCTCCCTACAAG | 151 | 99.2 |
