# Supplementary material for: Transcriptome Comparison Reveals Key Candidate Genes Responsible for the Unusual Reblooming Trait in Tree Peonies
Source: PLoS One. 2013 Nov 14;8(11):e79996. doi: 10.1371/journal.pone.0079996 (PMC3828231; doi:10.1371/journal.pone.0079996)
Supplement: Table S5 — Statistics of the mapping to all of the unigenes in the three floral inductions. (DOC) [file pone.0079996.s005.doc]

| **Table S5 Statistics of map to all unigenes in the three floral inductions** | | | | | | |
| --- | --- | --- | --- | --- | --- | --- |
| **Map to Gene** | **Reads number of L** | **Percentage** | **Reads number of H1** | **Percentage** | **Reads number of H2** | **Percentage** |
| Total reads | 12041487 | 100.00% | 12488008 | 100.00% | 12380315 | 100.00% |
| Total base pairs | 590032863 | 100.00% | 611912392 | 100.00% | 606635435 | 100.00% |
| Total mapped reads | 9773250 | 81.16% | 10212637 | 81.78% | 10080840 | 81.43% |
| Perfect match | 6351736 | 52.75% | 6989643 | 55.97% | 6855389 | 55.37% |
| Mismatch (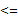2bp) | 3421514 | 28.41% | 3222994 | 25.81% | 3225451 | 26.05% |
| Unique match | 7035893 | 58.43% | 7278825 | 58.29% | 7226225 | 58.37% |
| Multi-position match | 2737357 | 22.73% | 2933812 | 23.49% | 2854615 | 23.06% |
| Total unmapped Reads | 2268237 | 18.84% | 2275371 | 18.22% | 2299475 | 18.57% |
