# Supplementary material for: Transcriptome Comparison Reveals Key Candidate Genes Responsible for the Unusual Reblooming Trait in Tree Peonies
Source: PLoS One. 2013 Nov 14;8(11):e79996. doi: 10.1371/journal.pone.0079996 (PMC3828231; doi:10.1371/journal.pone.0079996)
Supplement: Table S10 — The 21 genes putatively associated with floral induction in HN and LYH. (DOC) [file pone.0079996.s010.doc]

**Table S10 The 21 genes putatively associated with floral induction in HN and LYH**

| **Tree peony gene** | ***Arabidopsis* ID** | **Unigene ID** | **% identity with homologs** | **RPMK in H1**  **H1-VS-H2**  **FDR** | | | **RPMK in H2**  **L-VS-H1**  **FDR** | | **RPMK in L**  **L-VS-H2**  **FDR** |
| --- | --- | --- | --- | --- | --- | --- | --- | --- | --- |
| Autonomous pathway | | | | | | | | | |
| *PsFPA* | NP_181869.2 | CL2528.Contig1_All | 43 | 14.7  -0.23  0.19 | | | 12.5  -0.23  0.063 | | 17.2  -0.46  0.0001 |
| Ps*FVE* | NP565456.2 | CL3429.Contig1_All | 77 | 45.7  -0.23  0.26 | | | 39.1  0.34  0.02 | | 36  0.12  0.5 |
| *PsFY* | NP_196852.3 | CL4448.Contig1_All | 75 | 0.43  0.3  0.84 | | | 0.53  0.8  0.5 | | 0.25  1.1  0.26 |
| *PsFCA* | NP_850472.1 | CL2234.Contig7_All | 52 | 2.09  0.059  0.97 | | | 2.18  0.49  0.38 | | 1.49  0.55  0.19 |
| *PsLD* | CAJ53838.1 | CL9219.Contig1_All | 57 | 0.28  -8.12  0.41 | | | 0.001  -0.05  0.99 | | 0.27  -8.17  0.22 |
| Pohotoperiod pathway | | | | | | | | | |
| *PsCO* | NP_568863.1 | CL8074.Contig2_All | 47 | | 17.3  -0.47  0.027 | | 12.5  7.55  8.56E-54 | | 0.09  7.08  2.86E-38 |
| *PsGI* | NP_564180.1 | CL9113.Contig1_All | 75 | | 2.24  -0.03  0.98 | | 2.19  4.04  1.32E-14 | | 0.13  4.0  3.14E-14 |
| *PsPHYA* | NP_172428.1 | Unigene8157_All | 81 | | 36.6  -0.03  0.84 | | 35.6  0.92  6.72E-35 | | 19.3  0.89  2.77E-32 |
| *PsPHYB* | ABY61307.1 | CL3817.Contig2_All | 79 | | 4.77  -0.01  0.95 | | 4.74  0.93  3.00E-05 | | 2.5  0.92  3.75D-05 |
| *PsPIE1* | XP_002882790.1 | CL2502.Contig1_All | 74 | | | 26.7  -0.58  8.44E-07 | 17.9  0.6  1.58E-07 | 17.7  0.018  0.92 | |
| *PsCRY1* | NP_567341.1 | CL2628.Contig1_All | 79 | | | 30.3  -0.29  0.01 | 24.8  0.67  2.03E-11 | 19  0.39  0.0005 | |
| Vernalizaiton pathway | | | | | | | | | |
| *PsFRI* | NP_850923.1 | CL10369.Contig1_All | 63 | 48.3  -1.02  9.45E-29 | | | 23.8  -0.83  6.94E-36 | | 85.7  -1.85  1.1E-124 |
| Continuted | | | | | | | | | |
| *PsVIN3* | NP_200548.2 | Unigene3778_All | 41 | 12.76  0.75  8.11E-08 | | | 21.5  0.73  0.00017 | | 7.68  1.48  1.97E-20 |
| GA pathway | | | | | | | | | |
| *PsGA20OX* | NP_194272.1 | Unigene3745_All | 52 | 7.81  -1.17  0.00067 | | | 3.47  -0.35  0.013 | | 9.97  -1.52  1.67E-06 |
| *PsGID 1* | NP_187163.1 | Unigene2987_All | 81 | 243.4  -1.84  0 | | | 67.8  0.25  2.23E-10 | | 204.7  -1.6  5.26E-211 |
| *PsGA2OX* | NP_177965..1 | Unigene8928_All | 63 | 0.6  -9.23  0.4 | | | 0.001  1.54  0.53 | | 0.21  -7.72  0.61 |
| *PsRGA* | NP_178266.1 | Unigene13952_All | 62 | 120.99  -0.49  4.06E-22 | | | 86.45  0.96  3.11E-72 | | 62.32  0.47  2.43E-15 |
| Floral integrators and meristem identity genes | | | | | | | | | |
| *PsSOC1* | NP_182090.1 | Unigene15356_All | 61 | 19.1  1.4  2.64E-22 | | | 50.6  -1.33  2.2E-21 | | 48.2  0.067  0.63 |
| *PsFT* | NP_176726.1 | Unigene5093_All | 78 | 1.21  2.47  4.51E-06 | | | 6.73  1.95  0.15 | | 0.31  4.42  3.14E-10 |
| *PsLFY* | NP_200993.1 | CL5681.Contig1_All | 61 | 2.09  -2.57  0.0095 | | | 0.35  0.8  0.47 | | 1.2  -1.78  0.11 |
| *PsAP1* | NP_177074.1 | Unigene2945_All | 69 | 0.56  -0.31  0.76 | | | 0.45  1.27  0.44 | | 0.23  0.96  0.62 |

H1-VS-H2:log_2_ gene expression level in H1 compared to H2.

A FDR (false discovery rate) <0.001 indicates a significant difference.

Note: percent identity was calculated by comparing the *Arabidopsis* and peony sequences at the amino acid level.
